# Supplementary material for: Blocking ActRIIB and restoring appetite reverses cachexia and improves survival in mice with lung cancer
Source: Nat Commun. 2022 Aug 8;13:4633. doi: 10.1038/s41467-022-32135-0 (PMC9360437; doi:10.1038/s41467-022-32135-0)
Supplement: Supplementary file 1 — Supplementary Information [file 41467_2022_32135_MOESM1_ESM.pdf]

Supplementary information.

Blocking ActRIIB signaling and restoring appetite reverses cachexia and improves survival in mice with lung cancer

Andre Lima Queiroz[1,2*], Ezequiel Dantas[1,2*], Shakti Ramsamooj[1,2], Anirudh Murthy[1,2], Mujmmail Ahmed[1,2], Elizabeth R.M. Zunica[3], Roger J. Liang[1,2], Jessica Murphy[2,4,5], Corey D. Holman[6], Curtis J. Bare[6], Gregory Ghahramani[7], Zhidan Wu[8], David E. Cohen[6], John P. Kirwan[3], Lewis C. Cantley[2], Christopher L. Axelrod[3], and Marcus D. Goncalves[1,2,a]

[1] Division of Endocrinology, Department of Medicine, Weill Cornell Medicine, New York, NY 10065, USA.

[2] Meyer Cancer Center, Weill Cornell Medicine, New York, NY 10065, USA

[3] Pennington Biomedical Research Center, Baton Rouge, LA, 70808, USA

[4]Center for Molecular Oncology, Memorial Sloan Kettering Cancer Center, New York, NY 10065, USA

[5] Department of Pathology, Memorial Sloan Kettering Cancer Center, New York, NY 10065, USA

[6] Division of Gastroenterology and Hepatology, Department of Medicine, Weill Cornell Medicine, New York, NY 10065, USA

[7] Weill Cornell Graduate School of Medical Sciences, Weill Cornell Medicine, New York, NY 10065, USA

[8] Internal Medicine Research Unit, Pfizer Global R&D, Cambridge, MA, USA

*These authors contributed equally to this work.

[a]Corresponding author.

Supplementary Fig. 1

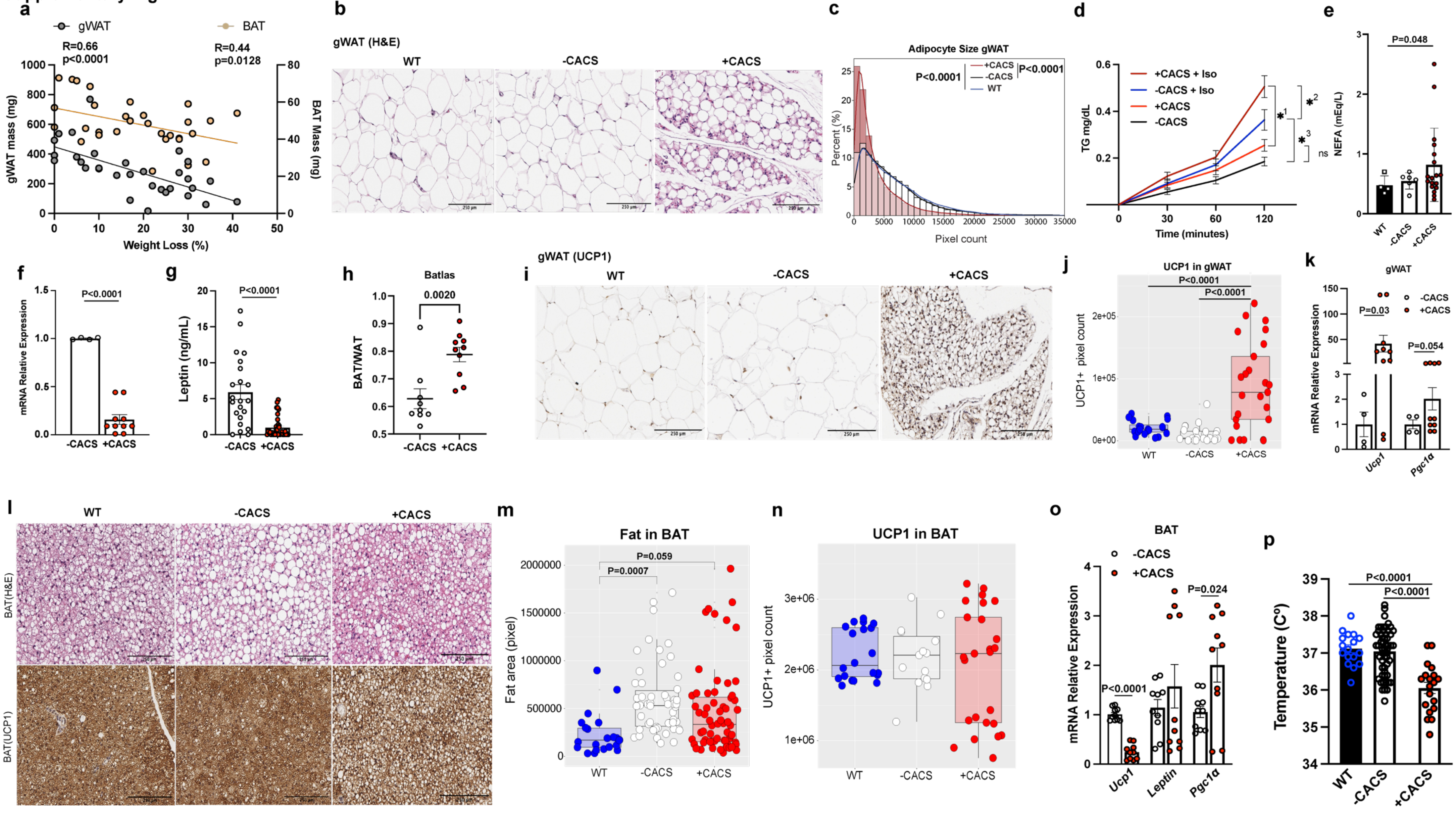

Fig. S1. CACS-induced WAT atrophy is associated with higher lipolysis a nd browning.

(a) Gonadal white adipose tissue (gWAT) and interscapular Brown adipose tiss ue (BAT) mass versus the percentage of total weight loss; **(b)** H&E staining of gonadal white adipose tissue (gWAT) from wild-type (left), non-cachectic (-CACS, center), and cachectic (+CACS, right) mice. **(c)** Quantification of gWAT adipocyte size in WT (n=4), -CACS (n=5) and +CACS (n=5). **(d)** Triacylglycerol (TG) release from dissected gWAT from -CACS (n=14), +CACS (n=19) mice following *ex vivo* incubation in the presence or absence of Isoproterenol (Iso) over a period of 120 minutes. **(e)** Non-esterified fatty acids (NEFA) measured in WT (n=4), -CACS (n=10) and +CACS (n=16) mice at the time of euthanasia. **(f)** Relative mRNA expression of *Lep* from gWAT of -CACS (n=4) and +CACS (n=10) mice. **(g)** Serum leptin levels from -CACS (n=21) and +CACS (n=36) mice at the time of euthanasia. **(h)** Deconvolution analysis of gWAT RNA-Seq to identify gene expression related to BAT signature in -CACS (n=9) and +CACS (n =10). **(i, j)** Uncoupling protein 1 (UCP1) immunohistochemistry (IHC) of the gWAT from representative WT (n=4), -CACS (n=5), and +CACS (n=5) mice and their relative UCP1+ positive pixel count quantifications per 20X microscopy field. **(k)** Relative mRNA expression of *Ucp1*, and *Ppargc1*α from gWAT from -CACS (n=4) and +CACS (n=10) mice. **(l)** Representative H&E (top) and UCP1 immunohistochemistry (bottom) staining of the BAT from WT, -CACS, and +CACS mice. **(m/n)** Quantification of fat area and UCP1+ staining from 5 high powered fields (20x) that were randomly selected from WT (n =4), -CACS (n= 5) and CACS (n=5) mice. **(o)** Relative mRNA expression of *Ucp1*, *Lep* and *Ppargc1*α from BAT from -CACS (n=10) and +CACS (n=10) mice. **(p)** Rectal temperature of WT (n = 17), -CACS (n = 56), +CACS (n= 19). Male and female mice were used in all panels. For boxplots in **j,m** and **n** horizontal bars within boxes represent medians. Tops and bottoms of boxes represent 25th and 75th percentiles, and vertical lines extend to the 1.5× interquartile range. Graphs show mean ± SEM. Pearson correlation analysis was used in a (R, Pearson r, and p, *P* value). Comparisons in f/g/h/k/o were made using two-tailed Student's t-test, and in (e) with one-tailed Student's t-test. Welch's correction for unequal standard deviation was used in **e,k** and **o**. Comparisons in (c) were made with two-sided Wilcoxon Rank Sum test. Comparisons in j/m/n/p were analyzed using one-way ANOVA and (d) with a two-way ANOVA followed by Tukey's multiple comparisons test (✱1<0.0001, ✱2<0.0115, ✱3<0.0006). Individual data points are independent biological replicates unless otherwise stated. Source data are provided as a Source Data file.

Supplementary Figure 2

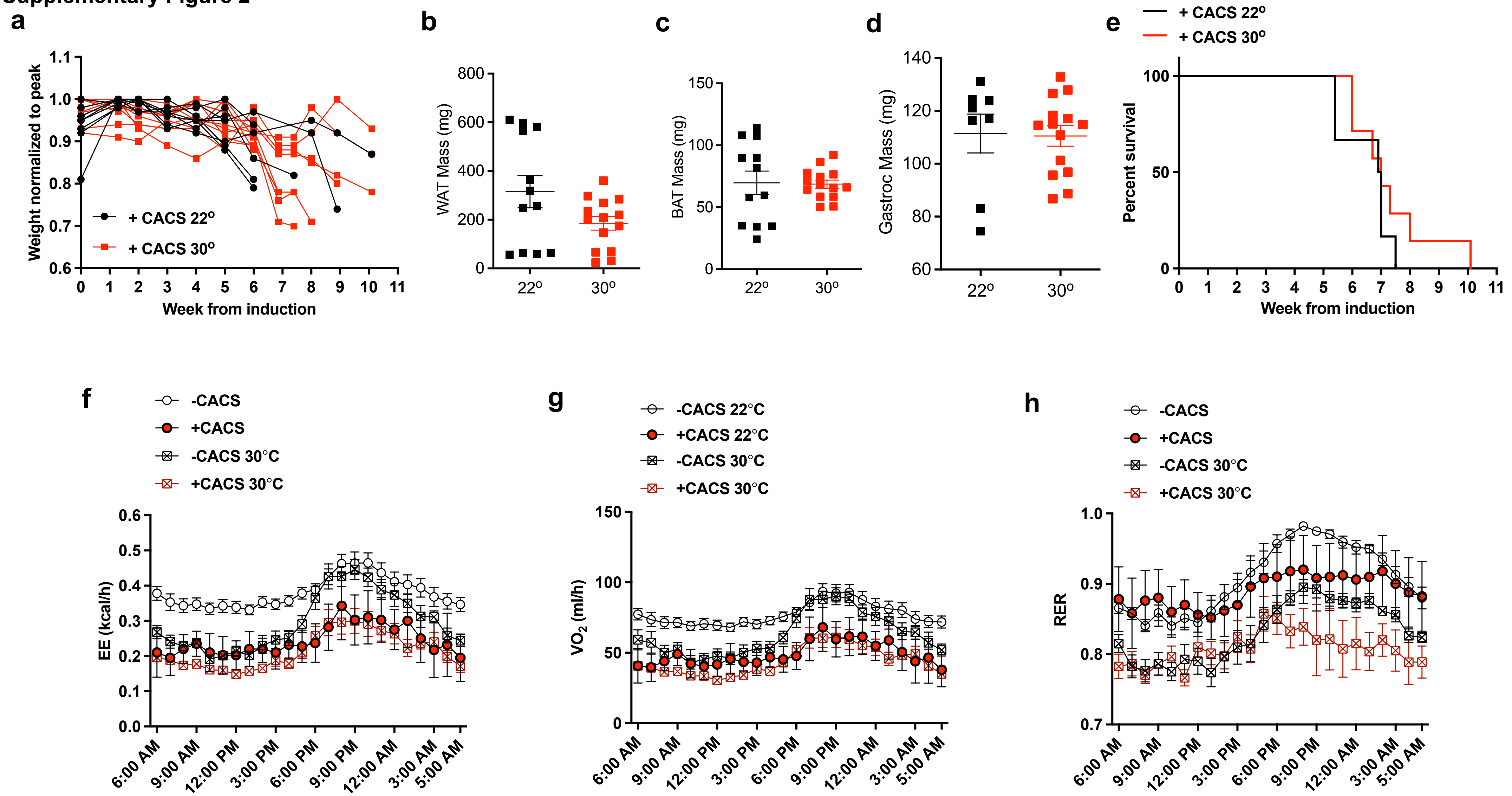

Fig. S2. Thermoneutrality does not improve cachexia. KL mice were induced with adeno-Cre and then four weeks later were randomized to an ambient temperature of 22°C (n = 9) or 30°C (n=11). **(a)** Weight normalized to the peak value following induction. The mass of the **(b)** gonadal white adipose tissue (WAT) (n=6/7 for 22/30°C), **(c)** scapular brown adipose tissue (BAT) (n=6/7 for 22/30°C), and **(d)** gastrocnemius muscle (n=5/7 for 22/30°C) are presented. **(e)** Percent survival over time of mice from A (n=9/11 for 22/30°C). **(f)** Energy expenditure in Kcal per hour, **(g)** volume of oxygen consumed by (ml/h) and **(h)** respiratory exchange ratio (RER) of -CACS and +CACS mice over a representative 24-hour period; Male and female mice were used in all panels. Graphs show mean ± SEM. Comparisons in b/c/d were performed using two- tailed Student's t-test. Kaplan Meier plot in (e) was assessed using Log-rank Mantel-Cox test. Comparisons in f/g/h were done using 2-way ANOVA. Individual data points are independent biological replicates unless otherwise stated. Source data are provided as a Source Data file.

Supplementary Figure 3.

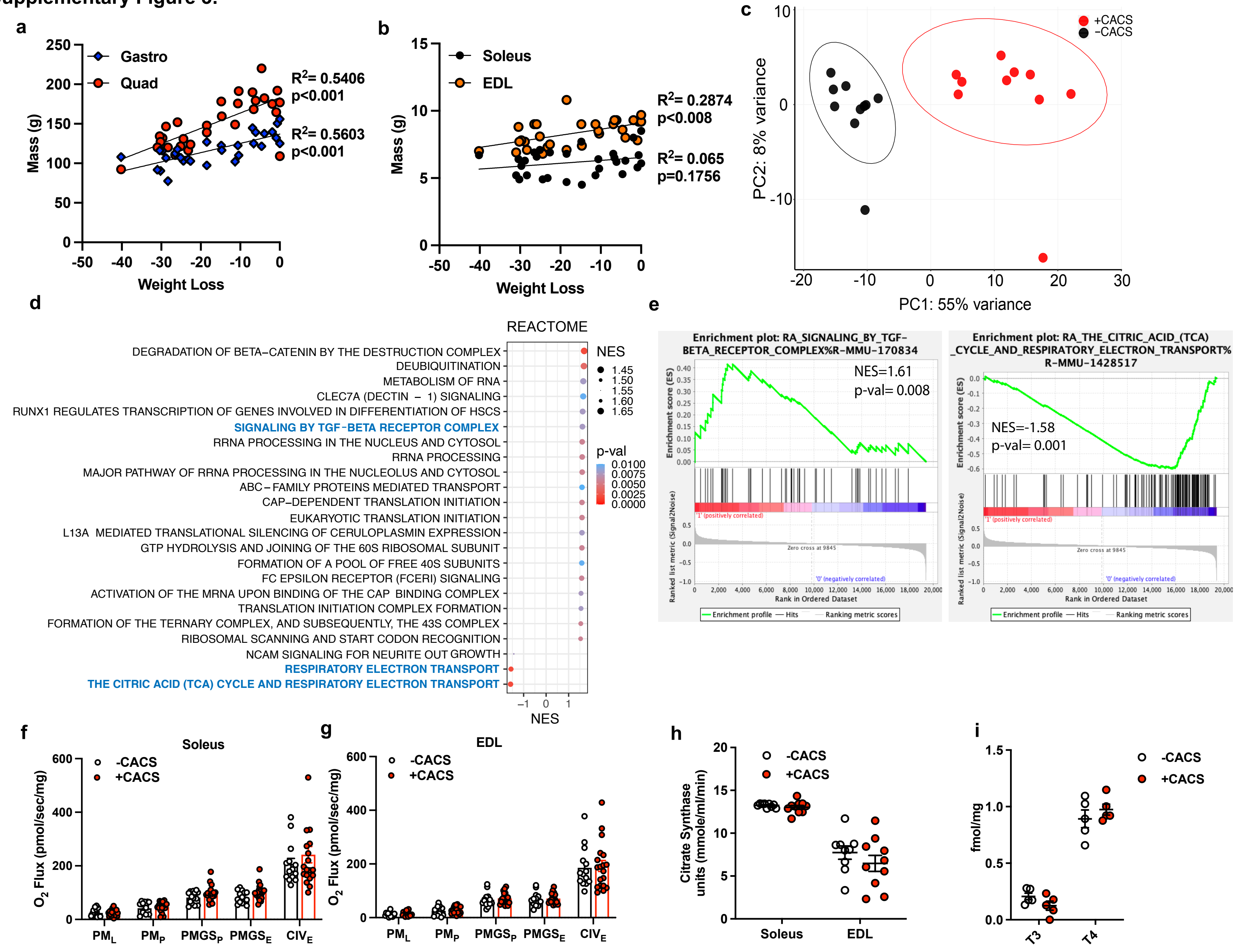

Fig. S3. Molecular and metabolic changes in the muscle of the cachectic mice.

(a) Gastrocnemius (n=30) and Quadriceps (n=30), and **(b)** Soleus (n=30) and EDL(n=30) mass of KL mice versus weight loss (% from peak weight). Correlation analysis (R^2, Linear regression, and *P* value). **(c)** Principal component analysis of gastrocnemius RNA-Seq data obtained from cachectic (+CACS, red, n=9) and non-cachexic (-CACS, black, n=10) mice; **(d)** Bubble plot depicting the most significant results from Gene Set Enrichment Analysis (GSEA) using the Reactome pathway database. **(e)** Enrichment plots for the Reactome "Signaling by TGF-β receptor complex" and "The citric acid (TCA) cycle and respiratory electron transport" pathways. **(f-g)** Oxygen consumption rates for permeabilized fibers obtained from Soleus (f), and EDL (g) of -CACS (n=14) and +CACS (n=19) mice. Oxygen flux due to mitochondria leak is denoted by subscript "L", NADH-linked flux is denoted by "P", and maximal flux in the presence of the mitochondrial uncoupler FCCP is denoted by "E". These rates were measured in the presence of pyruvate and malate (PM) and absence of ADP, and PM with the addition of ADP, glutamate and succinate (PMGS). Maximal oxygen flux through complex IV (CIV_E) was measured in the presence of TMPD and ascorbate. **(h)** Citrate synthase activity measured in soleus and EDL muscles of mice with(n=10) and without CACS (n=9); **(i)** T3 and T4 levels measured in gastrocnemius muscle of mice with (n=5) or without CACS (n=5). Male and female mice were used in panels a/b/f/g/h and (i). Only male mice were used in d/e. Data are shown as the mean ± SEM. Panels (f) and (g) were assessed by two-way ANOVA followed by Tukey's multiple comparisons test. Pearson correlation analysis was used in (a) and (b) (R^2, Linear regression, and p, *P* value). Individual data points are independent biological replicates unless otherwise stated. Source data are provided as a Source Data file.

Supplementary Figure 4

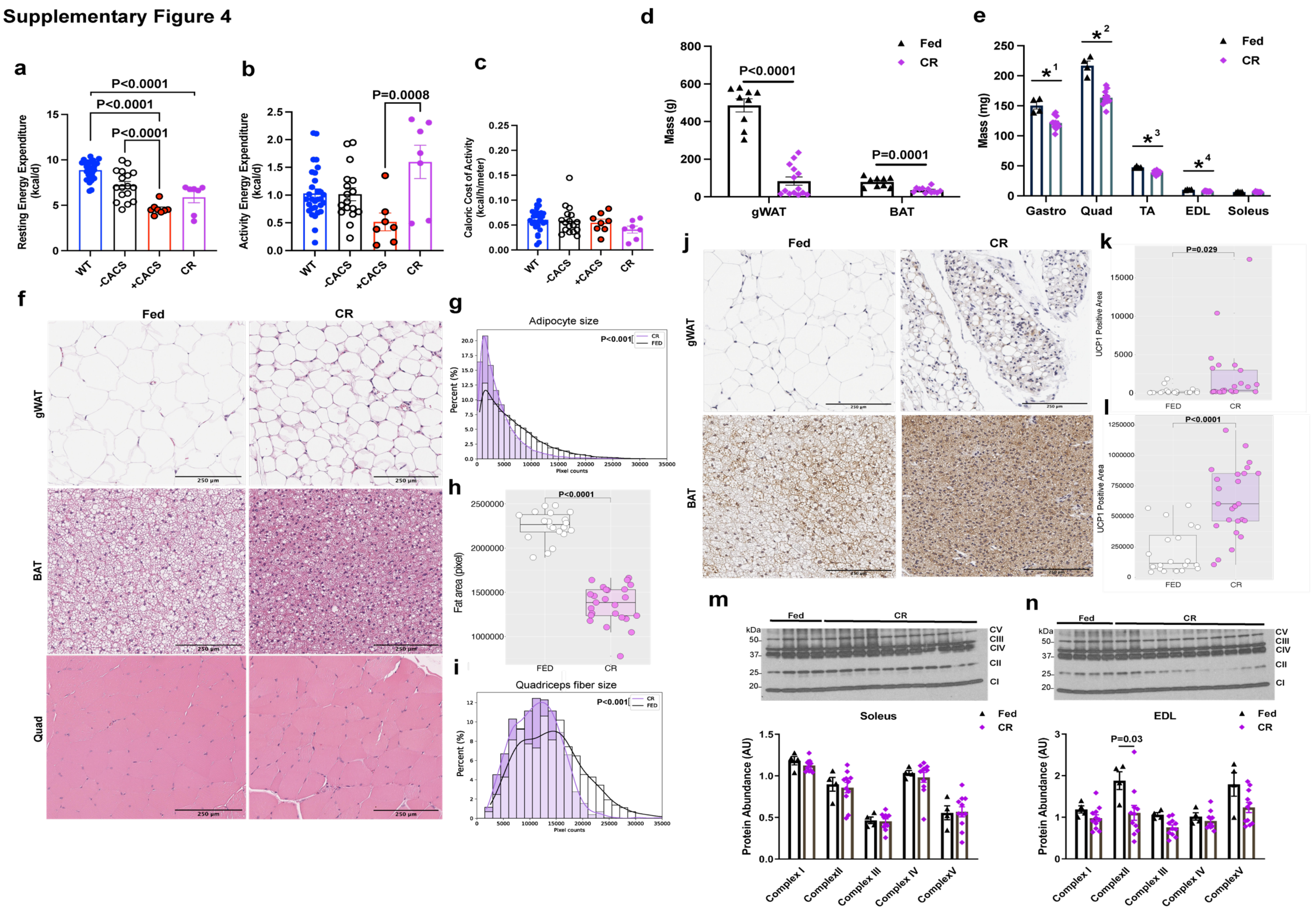

Fig. S4. The phenotype of the muscle, WAT and BAT is distinct in CR and CACS.

Wild-type mice were calorie restricted (CR) by feeding a ~8 kcal/day diet (amount consumed by cachectic mice) until weight stabilization. **(a)** Daily Resting energy expenditure; **(b)** Daily Activity energy expenditure; **(c)** Caloric cost of activity. **(d)** Gonadal white adipose tissue (gWAT) of WT (Fed, n=9) or CR (n=14) mice and interscapular brown adipose tissue (BAT) mass of WT (n=9) and mice following CR(n=11); **(e)** Gastrocnemius (Fed, n= 4 and CR, n=12), quadriceps (Fed, n=4 and CR, n=12), tibialis anterior (Fed, n=4 and CR, n=12), extensor digitorum longus (Fed, n=4 and CR, n=12), and Soleus muscle(Fed, n=4 and CR, n=12) wet mass (mg) at the time of euthanasia; **(f)** H&E staining of gWAT, BAT and Quad of Fed and CR mice. **(g)** Quantification of adipocyte size from Fed (n=4) and CR (n=5) mice. **(h)**Quantification of fat area in BAT from Fed (n=4) and CR (n=5) mice; **(i)** Quantification of quadriceps fiber size for Fed (n=4) and CR(n=5) mice. **(j)** Uncoupling protein 1 (UCP1) immunohistochemistry staining of a representative gWAT (top) and BAT (botton) from Fed and CR mice. **(k)** Quantification of UCP1 positive area in gWAT for Fed (n=4) and CR (n=5) mice; **(l)** Quantification of UCP1 positive area in BAT for Fed (n=4) and CR (n=5) mice. **(m)** Western blot analysis of mitochondrial oxidative phosphorylation complexes (CI-subunit NDUFB8, CII-SDHB, CIII-UQCRC2, CIV-MTCO1, and CV-ATP5A) in Soleus and **(n)** EDL muscles of Fed (n=4) and CR(n=11) mice. Alterations in energy expenditure-related parameters in WT, -CACS, +CACS, and CR mice. All CR and Fed mice in are wild-type male mice. -CACS and +CACS in a/b/c include both male and female mice. For the quantifications in g/h/i/k/l, 5 random high magnification microscopy fields (20X) were used per mouse. For boxplots in **h**, **k** and **l** horizontal bars within boxes represent medians. Tops and bottoms of boxes represent 25th and 75th percentiles, and vertical lines extend to the 1.5× interquartile range Graphs show mean ± SEM. a/b/c comparisons were made using one-way ANOVA followed by Tukey's multiple comparisons test. d/e(✱1=0.0003, ✱2<0.0001, ✱3<0.0001 and ✱4<0.0001). h/k/l/m/n comparisons were made using two-tailed Student's t-test compared with Fed mice. **g** and **i** were analyzed using a two-sided Kolmogorov-Smirnov test. Individual data points are independent biological replicates unless otherwise stated. Source data are provided as a Source Data file.

Supplementary Fig. 5

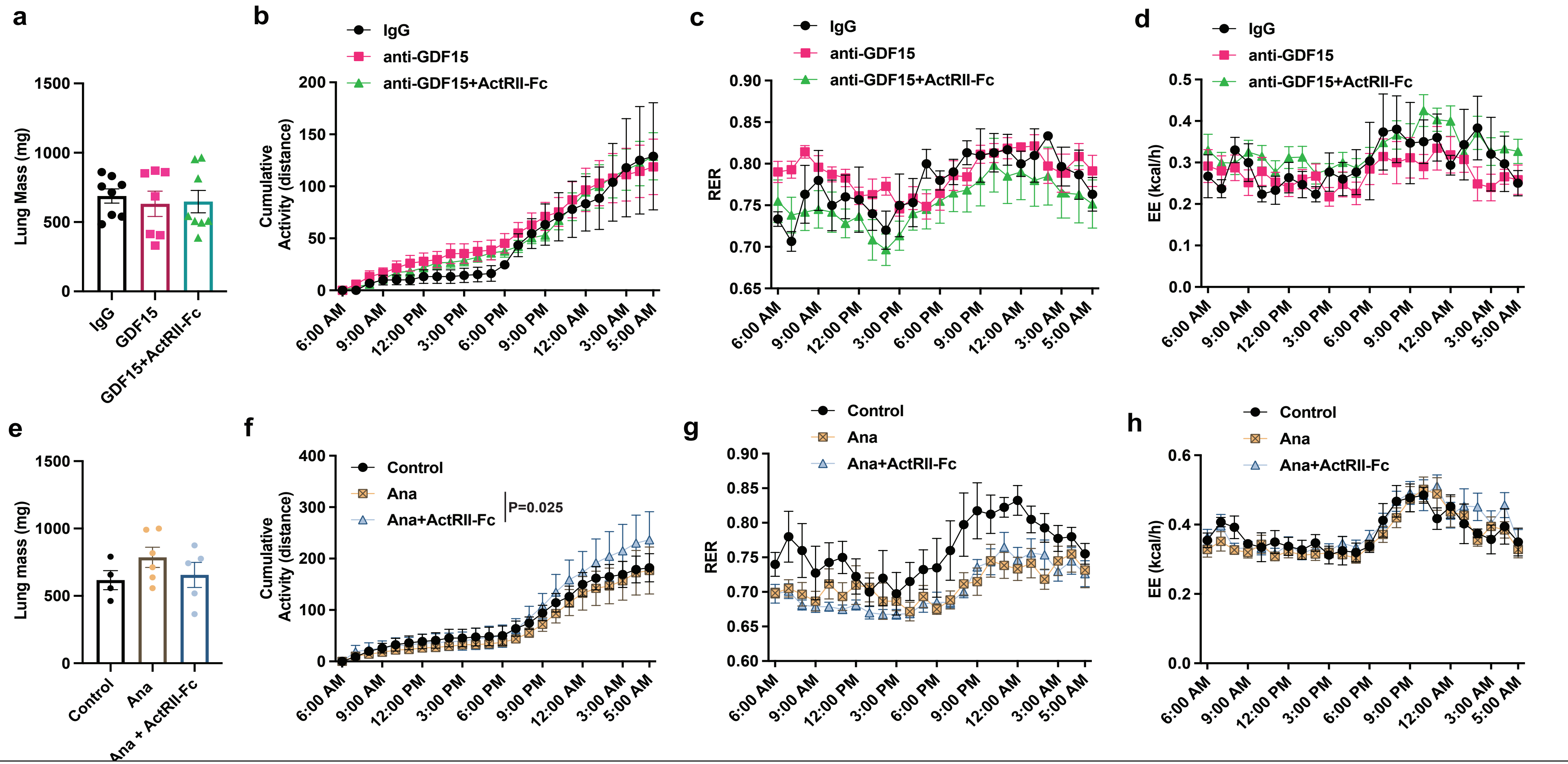

Fig. S5. ActRIIB-Fc in combination with Anamorelin but not anti GDF15 improves cumulative activity.

(a) Lung mass, **(b)** cumulative activity in meters, **(c)** respiratory exchange ratio (RER) and **(d)** energy expenditure (EE) over a representative period of 24 hrs in mice with cachexia (CACS) treated with either control immunoglobulin (IgG, n=8), anti-GDF15 monoclonal antibody (mAb, n= 7), or anti-GDF15 mAb together with a decoy ActRIIB-Fc mAb (n=8) for a maximum of 4 weeks. **(e)** Lung mass, **(f)** cumulative activity in meters,**(g)** respiratory exchange ratio (RER) and **(h)** energy expenditure (EE) over a representative period of 24 hrs in mice with cachexia (CACS) treated with either control immunoglobulin (IgG, n=8), anti-GDF15 monoclonal antibody (mAb, n= 7), or anti-GDF15 mAb together with a decoy ActRIIB-Fc mAb (n=8). Both male and female mice were used in panels **a-d**. Only female mice were used in **e-h**. Graphs show mean ± SEM. a/e comparisons were made using one-way ANOVA and b/c/d/f/g/h comparisons were made by 2-way ANOVA compared to IgG or Control followed by Tukey's multiple comparisons test. Individual data points are independent biological replicates unless otherwise stated. Source data are provided as a Source Data file.

Supplementary Figure 6

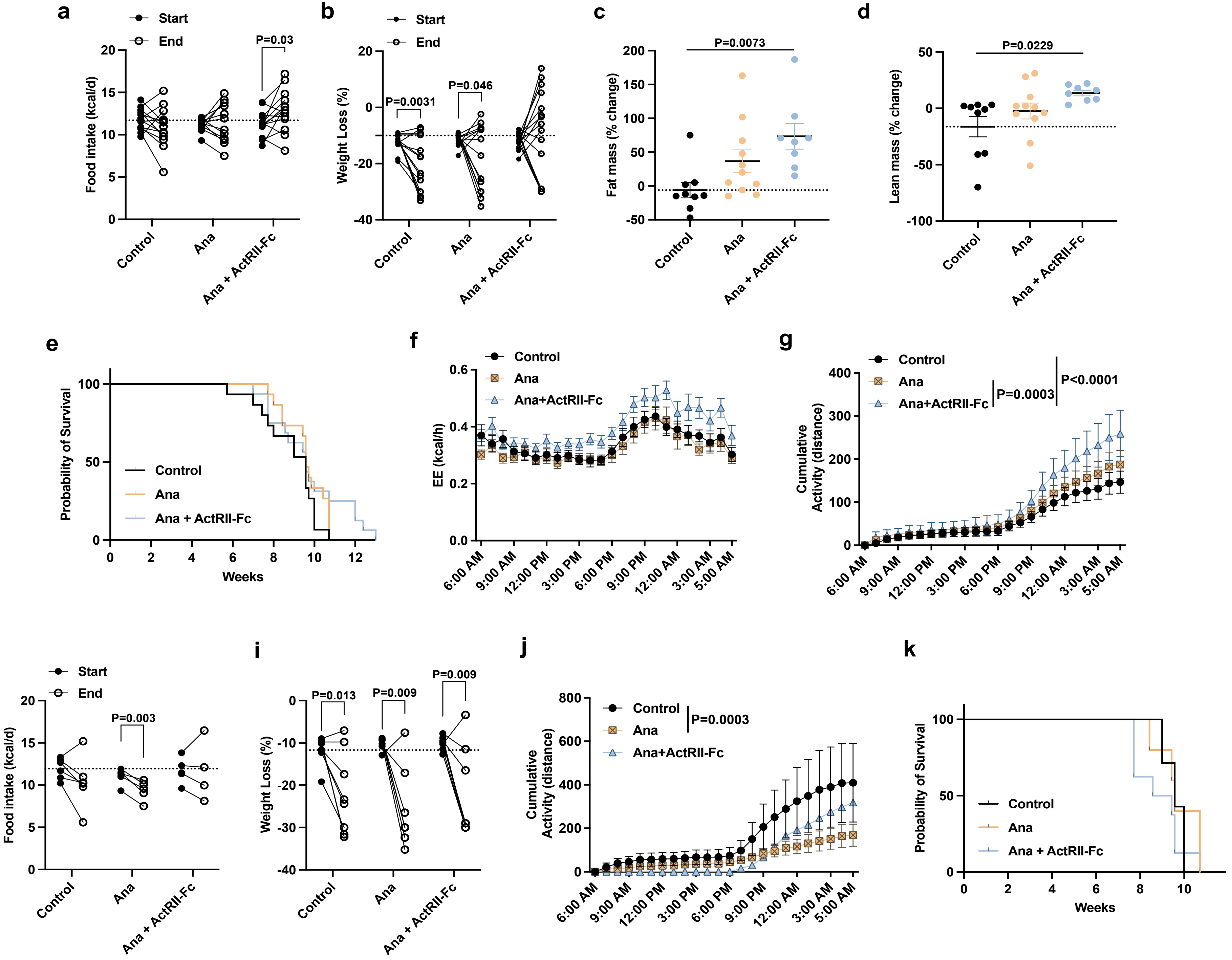

Fig. S6. The combination of Ana and ActRIIB-Fc shows no benefit in male mice.

(a) Food intake (kcal/d) at week 0 (Start) and after 2 weeks of treatment (End) with control vehicle (n=13), anamorelin (Ana, n=12) or ActRIIB-Fc decoy mAb in combination with Ana (n= 12). **(b)** Total body weight loss at Start and End in male and female mice treated with control (n=13), Ana (n=13) or Ana in combination with ActRIIB-Fc decoy mAb (n=13). Percentage of **(c)** fat mass and **(d)** lean mass change after 2 weeks of treatment with either control (n=9), Ana (n=11) or Ana in combination with ActRIIB-Fc decoy mAb (n=8) in both male and females. **(e)** Kaplan-Meier (KM) plot with the probability of survival of mice from (b). **(f)** Energy expenditure (EE) and **(g)** cumulative activity in meters over a representative period of 24 hrs of control mice (n=10), Ana (n=9) and Ana in combination with a decoy ActRIIB-Fc mAb (n=9) for 2 weeks. **(h)** Food intake (kcal/d) at week 0 (Start) and after 2 weeks of treatment (End) with control vehicle (n=6), anamorelin (Ana, n=5) or ActRIIB-Fc decoy mAb in combination with Ana (n= 4) on male mice. **(i)** Total body weight loss at Start and End in male mice treated with control (n=8), Ana (n=7) or Ana in combination with ActRIIB-Fc decoy mAb (n=8). **(j)** Cumulative activity in meters over a representative period of 24 hrs of male control mice (n=5), Ana (n=4) and Ana in combination with a decoy ActRIIB-Fc mAb (n=1) for 2 weeks. **(k)** Kaplan-Meier (KM) plot with the probability of survival of male mice with control vehicle(n=7), Ana(n=5) or Ana in combination with ActRIIB-Fc decoy mAb(n=8). Both male and female mice are represented in panels **a-g**. Only male mice are used in **h-k**. Graphs show mean ± SEM. a/b/h/i comparisons were made by two-sided paired Student’s t-test comparing “Start” to “End” for each treatment. c/d was evaluated by one-way ANOVA followed by Tukey’s multiple comparisons test. (e) and (k) were analyzed using Log-rank Mantel-Cox test. f/g/j were compared using 2-way ANOVA followed by Tukey’s multiple comparisons test. Individual data points are independent biological replicates unless otherwise stated. Source data are provided as a Source Data file.

Supplementary Figure 7

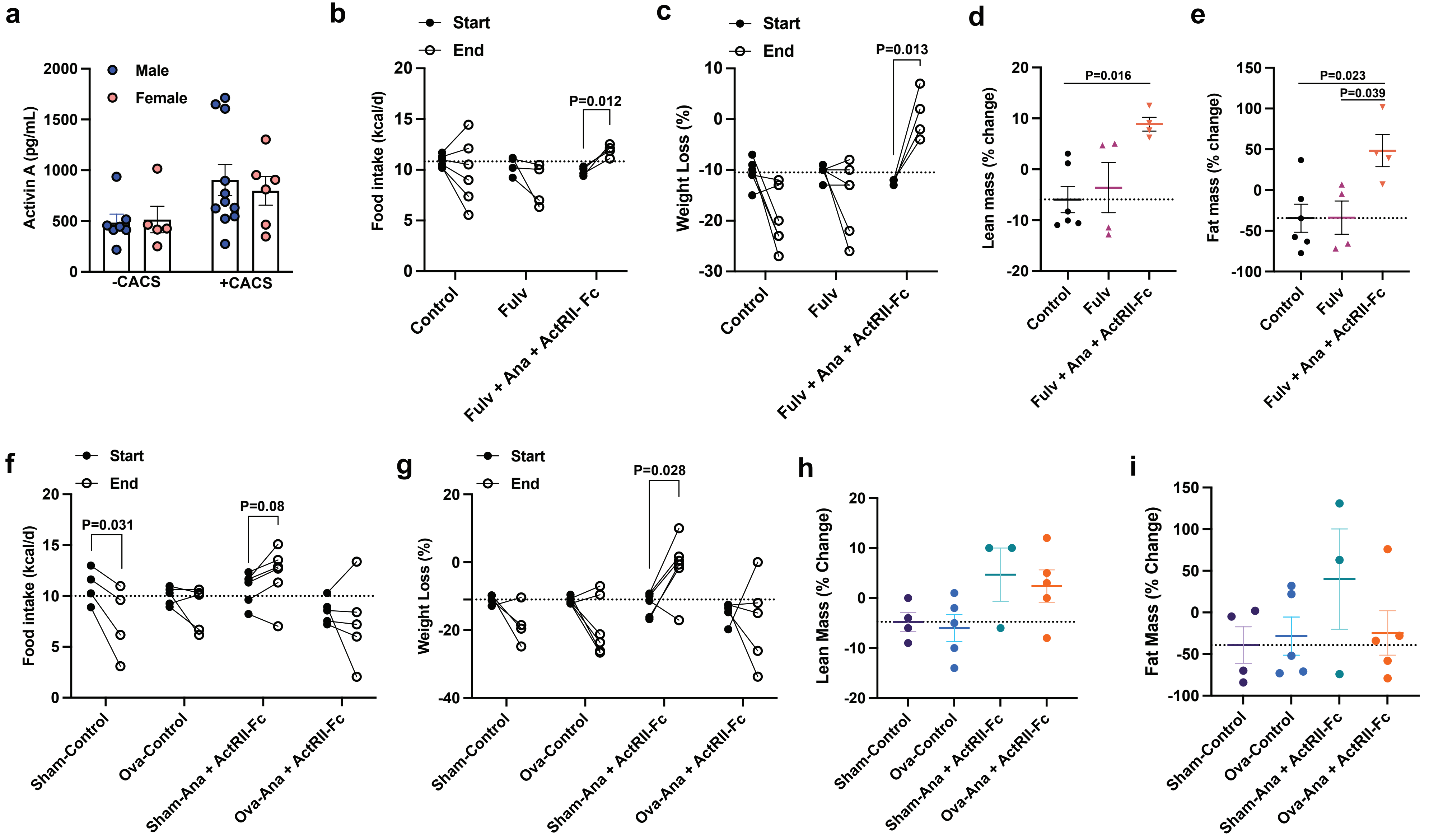

Fig. S7. Ovariectomy but not estrogen receptor inhibition prevents the beneficial effects of the combination therapy.

(a) Serum levels of Activin A stratified by sex in mice without (-CACS, n=12) and with CACS (+CACS, n=15) mice. **(b)** Food intake of female control mice (n=6), fulvestrant treated mice (n=4), and mice treated with fulvestrant in combination with Ana and ActRIIB-Fc decoy mAb (n =4). **(c)** Weight loss of female control mice (n=6), fulvestrant treated mice (n=4), and mice treated with fulvestrant in combination with Ana and ActRIIB-Fc decoy mAb (n=4). Change in **(d)** lean mass and **(e)** fat mass of control mice (n=6), fulvestrant treated mice (n=4), and mice treated with fulvestrant in combination with Ana and ActRIIB-Fc decoy mAb (n=4). **(f)** Food intake of female mice that underwent sham surgery or ovariectomy and were then randomized to receive vehicle (Sham-Control, n=4; Ova-Control, n=5), or combination therapy with Ana and ActRIIB-Fc decoy mAb (Sham-Ana+ActRIIB-Fc, n=6; Ova- Ana + ActRIIB-Fc, n=5). **(g)** Weight loss of female mice that underwent sham surgery or ovariectomy and were then randomized to receive vehicle (Sham-Control, n=4; Ova-Control, n=6) or combination therapy with Ana and ActRIIB-Fc decoy mAb (Sham-Ana+ActRIIB-Fc, n=6; Ova- Ana + ActRIIB-Fc, n=5). Change in **(h)** lean mass and **(i)** fat mass of mice that underwent sham surgery or ovariectomy and were then randomized to receive vehicle (Sham-Control, n=4; Ova-Control, n=5) or combination therapy with Ana and ActRIIB-Fc decoy mAb (Sham-Ana+ActRIIB-Fc, n=6; Ova-Ana + ActRIIB-Fc, n=5). Only female mice were used in panels **b-i**. Graphs show mean ± SEM. Comparison in (a) was done using unpaired two-tailed Student's t-test while comparisons in b/c/f/g were done using paired two-tailed t-test. d/e/h/i were analyzed using one-way ANOVA followed by Tukey's multiple comparisons test. Individual data points are independent biological replicates unless otherwise stated. Source data are provided as a Source Data file.
