# Supplementary material for: Blocking ActRIIB and restoring appetite reverses cachexia and improves survival in mice with lung cancer
Source: Nat Commun. 2022 Aug 8;13:4633. doi: 10.1038/s41467-022-32135-0 (PMC9360437; doi:10.1038/s41467-022-32135-0)
Supplement: Supplementary file 3 — Reporting Summary [file 41467_2022_32135_MOESM3_ESM.pdf]

nature portfolio

Corresponding author(s): Marcus D. Goncalves

Last updated by author(s): Jun 28, 2022

Reporting Summary

Nature Portfolio wishes to improve the reproducibility of the work that we publish. This form provides structure for consistency and transparency in reporting. For further information on Nature Portfolio policies, see our Editorial Policies and the Editorial Policy Checklist.

Statistics

For all statistical analyses, confirm that the following items are present in the figure legend, table legend, main text, or Methods section.

n/a	Confirmed	
☐	☒	The exact sample size (n) for each experimental group/condition, given as a discrete number and unit of measurement
☐	☒	A statement on whether measurements were taken from distinct samples or whether the same sample was measured repeatedly
☐	☒	The statistical test(s) used AND whether they are one- or two-sided<br>*Only common tests should be described solely by name; describe more complex techniques in the Methods section.*
☒	☐	A description of all covariates tested
☐	☒	A description of any assumptions or corrections, such as tests of normality and adjustment for multiple comparisons
☐	☒	A full description of the statistical parameters including central tendency (e.g. means) or other basic estimates (e.g. regression coefficient) AND variation (e.g. standard deviation) or associated estimates of uncertainty (e.g. confidence intervals)
☐	☒	For null hypothesis testing, the test statistic (e.g. F, t, r) with confidence intervals, effect sizes, degrees of freedom and P value noted<br>*Give P values as exact values whenever suitable.*
☒	☐	For Bayesian analysis, information on the choice of priors and Markov chain Monte Carlo settings
☒	☐	For hierarchical and complex designs, identification of the appropriate level for tests and full reporting of outcomes
☒	☐	Estimates of effect sizes (e.g. Cohen's d, Pearson's r), indicating how they were calculated

Our web collection on statistics for biologists contains articles on many of the points above.

Software and code

Policy information about availability of computer code

Data collection	STAR (v2.4.1d, 2-pass mode)
Data analysis	HTSeq (v0.6.1), R Studio (v3.6.3), DESeq2, DEBrowser

For manuscripts utilizing custom algorithms or software that are central to the research but not yet described in published literature, software must be made available to editors and reviewers. We strongly encourage code deposition in a community repository (e.g. GitHub). See the Nature Portfolio guidelines for submitting code & software for further information.

Data

Policy information about availability of data

All manuscripts must include a data availability statement. This statement should provide the following information, where applicable:

- Accession codes, unique identifiers, or web links for publicly available datasets
- A description of any restrictions on data availability
- For clinical datasets or third party data, please ensure that the statement adheres to our policy

All data to understand and assess the conclusion of this research are available in the main text, supplementary materials, Source Data file or GEO Database (accession number GSE165856). All available reagents are available from the Lead Contact under a material transfer agreement with Weill Cornell Medicine.

Field-specific reporting

Please select the one below that is the best fit for your research. If you are not sure, read the appropriate sections before making your selection.

- [x] Life sciences
- [ ] Behavioural & social sciences
- [ ] Ecological, evolutionary & environmental sciences

For a reference copy of the document with all sections, see nature.com/documents/nr-reporting-summary-flat.pdf

Life sciences study design

All studies must disclose on these points even when the disclosure is negative.

Sample size	Sample sizes were determined using G*Power version 3.1.9.7 for sample size calculation and based on previous experience in our group (sudden death was expected even before the start of treatment).
Data exclusions	No data were excluded from the analysis.
Replication	Experimental findings were verified by using multiple mice. Replications were successful.
Randomization	Mice were randomized in blocks of 6 and stratified by gender.
Blinding	Indirect calorimetry, RNAseq analysis, Metabolomic profiling, Histology, and IHC were performed in a blinded manner.

Reporting for specific materials, systems and methods

We require information from authors about some types of materials, experimental systems and methods used in many studies. Here, indicate whether each material, system or method listed is relevant to your study. If you are not sure if a list item applies to your research, read the appropriate section before selecting a response.

Materials & experimental systems

n/a	Involved in the study	
	x	Antibodies
x		Eukaryotic cell lines
x		Palaeontology and archaeology
	x	Animals and other organisms
x		Human research participants
	x	Clinical data
x		Dual use research of concern

Methods

n/a	Involved in the study	
x		ChIP-seq
x		Flow cytometry
x		MRI-based neuroimaging

Antibodies

Antibodies used	CP1 (ab209483); PGC1 alpha (ab54481); LDHA (CST #2012); Phospho-LDHA (Tyr10) (CST #8176); Pyruvate Dehydrogenase (CST #3205); Anti-Pdhe1α (Ser293) (ab92696); Total OXPHOS Cocktail (ab110413); GAPDH (Proteintech 10494-1-AP), VDAC (CST #4661); and α-Tubulin (DM1A) (CST #3873)
Validation	All antibodies were used in accordance to the manufacturer guidelines and confirmed to have previously been documented in the literature through CiteAb (www.citeab.com). UCP1 antibody was validated using tissues from UCP1-KO mouse published on abcam website.

Animals and other organisms

Policy information about studies involving animals; ARRIVE guidelines recommended for reporting animal research

Laboratory animals	10 -20 week old male and female KrasG12D/+; Lkb1f/f mice on a FVB background were used.
Wild animals	n/a
Field-collected samples	n/a
Ethics oversight	All animal studies were approved by the Institutional Animal Care and Use Committee (IACUC) of Weill Cornell Medical College and maintained as approved by the Institutional Animal Care and Use Committee (IACUC) at Weill Cornell Medicine (NY) under protocol number 2013-0116.

Note that full information on the approval of the study protocol must also be provided in the manuscript.

Clinical data

Policy information about clinical studies

All manuscripts should comply with the ICMJEguidelines for publication of clinical research and a completedCONSORT checklist must be included with all submissions.

Clinical trial registration	Human serum samples and clinical demographics were obtained from the IRB approved protocol, "The Validation of Serum Biomarkers for Lung-Cancer Induced Cachexia" (WCM IRB #19-11021135)
Study protocol	protocol can be obtained from the lead contact upon request.
Data collection	Clinical demographics and serum were collected after consent in the Weill Cornell Thoracic Surgery oncology practice.
Outcomes	The measurement of Activin A was an exploratory endpoint. Levels were measured using ELISA.
